# Cross-fostering immediately after birth induces a permanent microbiota shift that is shaped by the nursing mother

**DOI:** 10.1186/s40168-015-0080-y

**Published:** 2015-04-25

**Authors:** Joseph G Daft, Travis Ptacek, Ranjit Kumar, Casey Morrow, Robin G Lorenz

**Affiliations:** Department of Pathology, University of Alabama at Birmingham, 1825 University Blvd, SHEL 602, Birmingham, AL USA; Comprehensive Diabetes Center, University of Alabama at Birmingham, 1825 University Boulevard, SHEL 1207, Birmingham, AL USA; Department of Cell, Developmental, and Integrative Biology, University of Alabama at Birmingham, 1918 University Boulevard, Birmingham, AL USA; Department of Microbiology, University of Alabama at Birmingham, 3201 1st Avenue North, Birmingham, AL USA; Center for Clinical and Translational Science, University of Alabama at Birmingham, 1924 7th Avenue South, Birmingham, AL USA

**Keywords:** Murine, Maternal, Microbiota, Fecal, Cross-fostering, Type 1 diabetes

## Abstract

**Background:**

Current research has led to the appreciation that there are differences in the commensal microbiota between healthy individuals and individuals that are predisposed to disease. Treatments to reverse disease pathogenesis through the manipulation of the gastrointestinal (GI) microbiota are now being explored. Normalizing microbiota between different strains of mice in the same study is also needed to better understand disease pathogenesis. Current approaches require repeated delivery of bacteria and large numbers of animals and vary in treatment start time. A method is needed that can shift the microbiota of predisposed individuals to a healthy microbiota at an early age and sustain this shift through the lifetime of the individual.

**Results:**

We tested cross-fostering of pups within 48 h of birth as a means to permanently shift the microbiota from birth. Taxonomical analysis revealed that the nursing mother was the critical factor in determining bacterial colonization, instead of the birth mother. Data was evaluated using bacterial 16S rDNA sequences from fecal pellets and sequencing was performed on an Illumina Miseq using a 251 bp paired-end library.

**Conclusions:**

The results show that cross-fostering is an effective means to induce an early and maintained shift in the commensal microbiota. This will allow for the evaluation of a prolonged microbial shift and its effects on disease pathogenesis. Cross-fostering will also eliminate variation within control models by normalizing the commensal microbiota between different strains of mice.

**Electronic supplementary material:**

The online version of this article (doi:10.1186/s40168-015-0080-y) contains supplementary material, which is available to authorized users.

## Background

In recent years, it has been appreciated in both animal models and human patients that there are healthy and disease-promoting microbiota [1-3]. This can be seen in diseases such as inflammatory bowel disease (IBD) and type 1 diabetes (T1D) [4-8]. In order to study the effects of the microbiota in healthy and diseased subjects, research has focused on replacing or shifting disease-promoting microbiota to healthy microbiota, thus potentially reversing the diseased state. This has proven to be quite challenging because within healthy individuals there is considerable variation in the microbiota. For example, mice of the same strain, housed in different cages, have a diverse microbiota that can account for up to 30% of the variance seen in microbiome studies [9]. In an attempt to properly control the microbiota in animal models, antibiotics, gavage of fecal content, co-housing, or cross-fostering have all been used [10-14]. However, these experiments have multiple drawbacks, including the fact that they require repeated delivery of bacteria and a large number of animals because the microbiota already present is not easily displaced. Further confounding the field is the wide variance in microbiota between the same strains at different facilities. A well-published example is the presence of segmented filamentous bacteria (SFB) in C57BL/6 mice ordered from Jackson Labs that are absent in C57BL/6 mice ordered from Taconic Labs [15]. Differences can even be seen in colonies within the same facility [16,17].

Recent data has revealed the importance of maternal transmission of microbiota in the colonization of their offspring. Experiments with toll-like receptor (TLR) knockouts and wild-type mice born to TLR knockout mothers via a heterozygous breeding reveal an identical microbiota between all pups regardless of TLR status [18]. This finding contradicts the previous conclusion that TLR signaling plays a role in dictating the microbiota as shown by Wen *et al*. who showed a difference in the microbiota between MyD88^KO/+^ and MyD88^KO^ mice [19]. One potential reason for these differing results could be that these mice were derived from germfree mothers and bacteria were then introduced. Therefore, mice were not initially colonized by the maternal microbiota because mothers were germfree. This emphasizes that variation in microbiota maybe due to differences in the microbiota of the nursing mothers, not due to the knockout state of the pups. Controlling for colonization of maternal microbiota by using the same mother for all pups allows for proper controls when comparing different genotypes on the same genetic background. However, it does not address the proper way to compare microbiota between completely different strains that by definition have to be born to strain-specific mothers.

A second method to normalize bacterial colonization is co-housing. Co-housing can induce a change in the gut microbiota but requires one recipient (receiving new microbiota) to be housed with three donors (giving microbiota), requiring a large number of animals and a high experimental cost [10]. Another method that has been effectively used in mice to shift microbiota is the addition of fecal bacteria from diabetic-resistant MyD88-deficient mice to drinking water [11]. The administration of this fecal water for a period of 3 weeks to non-obese diabetic (NOD) mice causes an increase in *Lachnospiraceae* and *Clostridiaceae*, while leading to a decrease in *Lactobacillaceae*. This shift in microbiota correlates with a reduction in the incidence of diabetes, but this treatment cannot be started until mice are approximately 4 weeks of age [11]. Therefore, a better model is needed that is cost effective and that can manipulate the colonization of microbiota at birth.

In humans, microbiota shifts are being induced by diet or in the case of patients with *Clostridium difficile*, by fecal transplants. Dietary studies in humans have revealed that you can induce a shift in microbial diversity with a plant-based and animal-based diet. However, as soon as subjects are taken off of their respective diet, microbial diversity returns to pre-diet levels within days [12]. It is hypothesized that fecal transplants lead to colonization by bacteria that occupy the niche of *C. difficile* preventing it from colonizing the gut; however, the exact mechanism and long-term effects are still unknown [20-22].

Two problems that exist with current protocols are that the microbial shifts are not permanent and that shifts are not introduced prior to the development of the rest of the gastrointestinal (GI) ecosystem. To properly study the sustained efficacy of shifting the GI microbiota, a method must exist that induces a long-term shift early in life. Currently, it is hard to accurately determine the benefits of altering the composition of an individual’s microbiota if these shifts are not stable or if they are not introduced until later in life.

**Figure 1:**
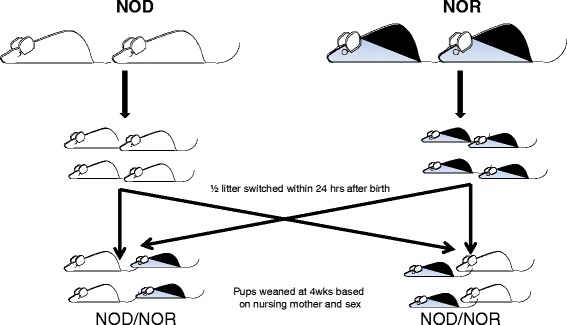
Experimental design of cross-fostering between mice of opposite strains. Breeding pairs of NOD and NOR mice are set up simultaneously. Pups that are born within 48 h of each other to their respective parent are switched to a nursing mother of a different strain. Only half of the litters are switched, leaving half of each original litter with their birth mother. As is standard for the weaning protocols in our animal facility, weaning pups are separated based on sex and nursing mother. Resulting cages will then contain mice of the same sex, but of mixed strains.

Methods currently used to induce microbial shifts in the GI system are often inefficient and ineffective. A method is therefore needed to induce a sustained microbial shift. We propose cross-fostering as a means of efficiently and effectively inducing a sustained microbial shift. To test this hypothesis, we designed an experiment that we believed would allow early colonization of mouse pups with maternal microbiota and we postulated that this microbiota would remain stable for the entire lifespan of the test subjects. The NOD and non-obese diabetic-resistant (NOR) strains of mice were used to explore whether it was possible to induce an early and permanent shift between different strains of mice. To induce a change in the microbiota as early as possible, newborn pups from NOD and NOR mothers were cross-fostered unto the opposing strains. Cross-fostering is the switching of newly born pups to non-birth mothers who themselves have recently had pups or are ready to nurse (Figure 1). The pups were nursed by mothers of the opposite NOD and NOR strains until weaning. At weaning, pups were separated based on sex, but not strain, and feces was collected from pups and mothers for microbiome analysis by sequencing of the 16S rDNA gene using next-generation sequencing (Illumina MiSeq; Illumina, San Diego, CA, USA). When the study ended at 32 weeks, feces were again collected from the previously cross-fostered mice for microbiome analysis. Comparison of bacterial phyla was then made between mice at weaning and the end of the study. This analysis of microbiota at 4 weeks and 32 weeks will determine if cross-fostering causes a microbial shift to resemble the nursing mother, and it will also determine if this shift is temporary or permanent.

## Results and discussion

### Nursing mother, not birth mother, determines fecal microbiota composition

**Figure 2:**
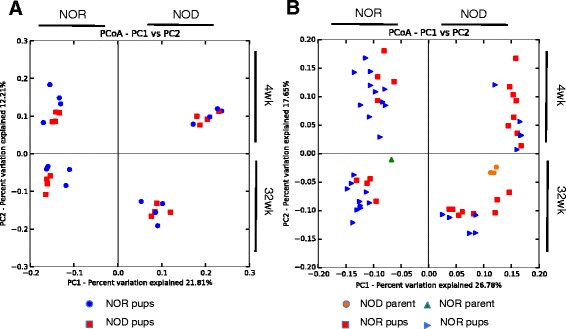
Grouping of fecal bacterial groups from mice nursed by a NOD or NOR mother, each quadrant is labeled with NOD or NOR, indicating nursing mother. PCoA plots were generated from bacterial DNA that was isolated from mouse fecal material and the V4 segment of the 16S rRNA gene was amplified from fecal pellets from mice nursed by NOD and NOR mothers. Group clustering represents a difference in β-diversity between mice nursed by NOD or NOR mothers. **(A**, **B)** Experiment 1 included NOD (*n* = 8) and NOR (*n* = 8) mice, and experiment 2 had NOD (*n* = 12) and NOR (*n* = 16) mice. Significant differences (*P* < 0.05) in beta diversity were calculated using compare_categories.py using the PERMANOVA test.

**Figure 3:**
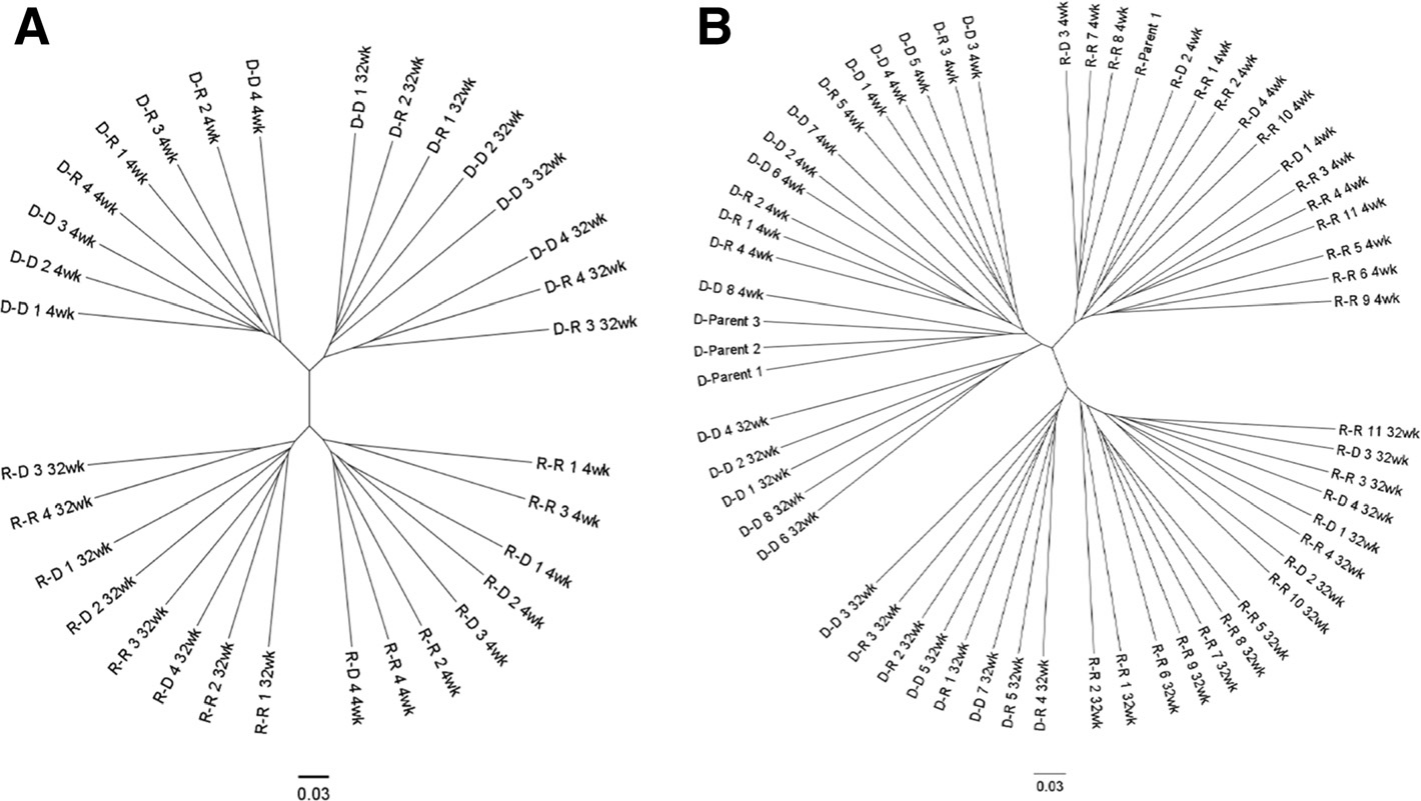
Phylogenetic tree of fecal bacteria from nice nursed by a NOD or NOR mother. Banding similarity analysis of the samples from NOD and NOR mice reveal that the highest degree of similarity exists between mice that were nursed by the same mother regardless of pup strain. **(A, B)** A high degree of similarity also exists between groups based on age with groupings at 4 and 32 weeks. The mice are identified with the following naming convention: Nursing Mother-Pup Strain (where R = NOR and D = NOD).

The relationships between microbial communities in NOD and NOR mice that had been nursed by either NOD or NOR mothers were visualized by phylogenetic analysis using principal component of analysis (PCoA) plots using the unweighted unifrac distance matrices (Figure 2). Four distinct groupings were seen based on nursing mother (not birth mother) and age. This was replicated in two separate experiments (experiment 1 and experiment 2) using unique parents in each experiment. The grouping is also clear when visualizing the distance matrix of the samples (which was used to generate the PCoA plots) as a phylogenetic tree (Figure 3). Both the PCoA plots and phylogenetic tree are visualizations of β-diversity, which was significantly different between all four clusters (*P* < 0.001 for clustering by age and by nursing mother). Clustering by nursing mother and by age was also significant when using the weighted unifrac distance matrix (*P* = 0.002 for both nursing mother and age, PCoA plot shown in Additional file 1). Significant statistical differences based on caging or in α-diversity between mice nursed by NOR or NOD mothers were not seen (*P* > 0.05, Additional files 2, 3). Feces from 4-week-old NOD and NOR pups nursed by a NOR mother have microbiota resembling that of NOR mice, while feces from 4-week-old NOD and NOR mice nursed by a NOD mother have microbiota resembling that of NOD mice. This is seen at weaning and at 32 weeks when the study ended. It is important to note that the groupings shift between 4 and 32 weeks, but even at 32 weeks, mice are still grouping based on nursing mother and not birth mother.

### Mice nursed by NOD and NOR mothers have different fecal microbiota

**Figure 4:**
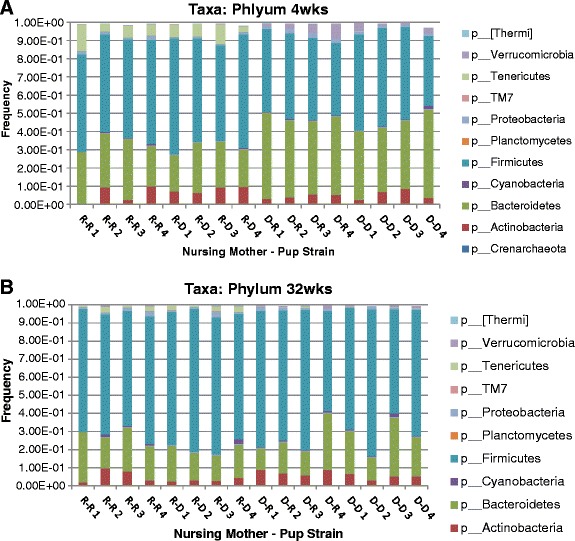
Phyla proportions in mice fostered by NOR and NOD mothers. These are stacked bar charts showing the cumulative proportions of bacterial phyla from NOR- and NOD-fostered mice. The mice are identified on the x axis with the following naming convention: Nursing Mother-Pup Strain (where R = NOR and D = NOD). **(A)** Proportions at 4 weeks of age. **(B)** Proportions at 32 weeks of age.

To compare the fecal microbiota of NOD- and NOR-fostered mice, we first examined the relative proportions of bacterial phyla in these mice (Figure 4). Because the samples showed significant differences in clustering by age, analyses were stratified by age. At 4 weeks, the average proportions of *Bacteroidetes*, *Firmicutes*, *Tenericutes*, *Verrucomicrobia*, and candidate division TM7 were significantly different in NOD- and NOR-nursed mice and at 32 weeks, the average proportions of *Tenericutes* and TM7 were significantly different. At both time points, *Tenericutes* and TM7 were higher in NOR-nursed mice, while at 4 weeks, *Firmicutes* were higher in NOR-nursed mice and *Bacteroidetes* were higher in NOD-nursed mice.

**Figure 5:**
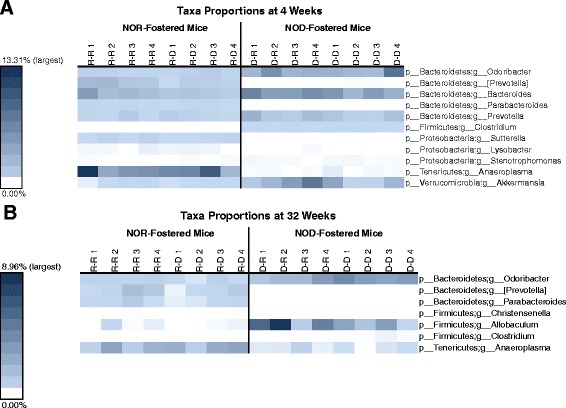
Significant differences in genera proportions between NOR- and NOD-fostered pups. This is a heatmap showing bacterial genera with statistically significant differences in proportion (*P* < 0.05 after FDR correction) between NOR- and NOD-fostered mice at 4 weeks of age. Color shading is based on the proportion of each genera within fecal samples from each mouse, with the darkest color being the highest observed proportion (by time point) and white being zero (that is, not present). Mouse ID is shown above each column with the following naming convention: Nursing Mother-Pup Strain (where R = NOR and D = NOD). **(A)** Differences at 4 weeks of age. **(B)** Differences at 32 weeks of age.

The quality of our sequencing allowed us to further resolve the differences in composition of fecal microbiota of NOD and NOR mice nursed by NOD mothers at the genus level. Genera with a statically significant difference by either ANOVA (difference in quantity) or G-test (difference in presence/absence) after false discovery rate (FDR) correction were selected. Like analyses at the phylum level, these analyses were stratified by age. At 4 weeks of age, NOR-nursed mice had higher proportions of *Prevotella*, *Parabacteroides*, *Sutterella*, *Lysobacter*, *and Anaeroplasma*, while NOD-nursed mice had higher proportions of *Odoribacter*, *Bacteroides*, *Prevotella*, *Clostridium*, *Stenotrophomonas*, and *Akkermansia* (Figure 5A). At 32 weeks of age, NOR-nursed mice had higher proportions of *Prevotella*, *Parabacteroides*, *Christenella*, and *Anaeroplasma*, while NOD-nursed mice had higher proportions of *Odoribacter*, *Allobaculum*, and *Clostridium* (Figure 5B). Note that [*Prevotella*], in both Figure 5A,B, is a provisional taxonomical assignment by Greengenes of operational taxonomic units (OTUs) different from canonical *Prevotella*.

### Changes of the fecal microbiota due to age differ by nursing mother

**Figure 6:**
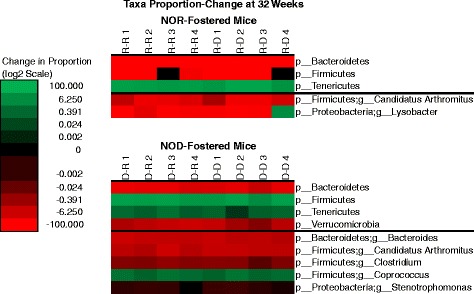
Significant changes in phyla and genera proportions from 4 weeks to 32 weeks of age in NOR- and NOD-fostered pups. This is a heatmap showing statistically significant changes in the proportions of phyla and genera (*P* < 0.05 after FDR correction) from 4 weeks of age to 32 weeks. NOR- and NOD-fostered pups are shown separately. Color shading is based on the absolute change in taxa proportion on a log-2 scale, with green indicating increases in proportion, red indicating decreases in proportion, and black indicating no change. Mouse ID is shown above each column with the following naming convention: Nursing Mother-Pup Strain (where R = NOR and D = NOD).

Because samples also clustered by age, differences in fecal microbiota by age were resolved. The largest differences between the two time points were visible at the phylum level, so statistical tests were run at the phylum and genus levels. Because samples clustered by nursing mother, analyses were stratified by nursing mother. From 4 weeks to 32 weeks of age, NOR-nursed mice had increases in the phylum *Tenericutes* and decreases in the phyla *Bacteroidetes* and *Firmicutes* and in the genus *Candidatus Arthromitus*. During the same period, NOD-fostered mice had increases in the phyla *Firmicutes* and *Tenericutes* and in the genus *Coprococcus*, and decreases in the phyla *Bacteroidetes* and *Verrucomicrobia* and in the genera *Bacterioides*, *C. Arthromitus*, *Clostridium*, and *Stenotrophomonas* (Figure 6).

### NOD mice fostered by NOR parents have a decreased incidence of T1D

**Figure 7:**
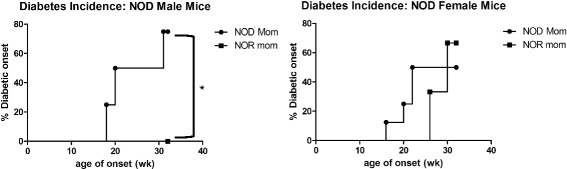
NOD mice fostered by NOR parents have a decreased incidence of T1D. Incidence of T1D in NOD male (left) and female (right) mice nursed by NOD (filled circle) or NOR (filled square) mothers. Data combined from two separate groups of cross-fostered pups. Significance was determined by the Mantel-Cox Text. *P = 0.0013 at 32 weeks of age.

In order to demonstrate that cross-fostering not only can impact the composition of fecal microbiota of the cross-fostered pups but can also have a functional impact on the animals, we followed all cross-fostered mice until 32 weeks of age to determine the incidence of diabetes. Mice were considered to have diabetes when they had a positive urine glucose test followed by blood glucose test of 200 mg/dL or greater. Cross-fostering of male NOD mice onto NOR parents was protective against the development of T1D, with no development of diabetes in NOD male mice nursed by a NOR mother, compared to approximately 80% disease incidence in male NOD mice nursed by NOD mothers (Figure 7). Interestingly, it has been shown that transfer of microbiota from male NOD mice, which have a higher resistance to disease development than do female mice, can lower disease incidence [23]. Although a similar trend towards decreased disease onset was seen in female NOD mice nursed by a NOR mother (Figure 7), this difference was not statistically significant (*P* = 0.16).

## Conclusions

Grouping based on bacterial sequences from fecal pellets of NOR and NOD mice revealed that the nursing mother, not the birth mother, dictates the composition of fecal microbiota. Not only are these groupings present at weaning (approximately 4 weeks) but remain throughout the lifetime of the mice (32 weeks). Our experimental approach included caging the mice after weaning in groups of four to five based on gender, as that is our standard experimental housing for our T1D experiments. This does introduce the concern that there could be an impact of caging on the fecal microbiota of these pups as they age. Our analysis did not indicate a significant impact of caging on the microbial content; however, we did not confirm our data through any experiments on individually caged pups. All significant alterations in the fecal microbiota that we observed were attributable to the nursing parent microbiota. Altering the microbiota to improve disease incidence has been previously achieved in the NOD mouse through the gavage of cecal contents from male mice into female mice. In these studies, the gavage of cecal contents was not started until weaning and changes in microbiota were measured at 14 and 34 weeks of age [23]. These results do not indicate the effects of inducing a microbiota shift earlier in life or to what extent the initial cecal gavage at 6 weeks is altering the microbiota. Ubeda *et al*. have previously shown that the composition of microbiota in mice is largely determined by maternal transfer to pups regardless of genetic background, although they did not look at the cross-fostering of two different strains [18]. Cross-fostering experiments after Cesarean section indicate the importance of the ability of the nursing mother to dictate the microbial composition over birth mothers [24]. However, the ability to cause a long-lasting microbial shift after vaginal birth is not addressed.

When focusing on T1D, cross-fostering brings to light some interesting trends. In children with T1D, the *Firmicutes* to *Bacteroidetes* ratio was significantly lower when compared to age-matched healthy children [25]. During cross-fostering, mice nursed by a NOD mother and mice nursed by NOR mothers had a similar ratio of *Firmicutes* and *Bacteroidetes* as that seen in diabetic compared to healthy control in both mice and humans, with a higher ratio of *Firmicutes* to *Bacteroidetes* seen in mice nursed by a diabetic-resistant NOR mother [5]. The role of the other bacterial phylum (*Verrucomicrobia*, TM7, and *Tenericutes*) that are significantly different between mice nursed by NOD or NOR mothers in the context of T1D is not yet known. Looking beyond the phylum level, mice nursed by a NOD mother were positive for *Clostridium*, while mice nursed by a NOR mother were negative. This data mirrors what is seen in human subjects in which *Clostridium* levels are higher in diabetic children compared to healthy children [25].

In our analyses, there were two taxonomical groups identified as *Prevotella*: one having the canonical *Prevotella* sequence, and one having a non-canonical sequence provisionally assigned to *Prevotella* by Greengenes (indicated as [*Prevotella*]). In mice nursed by NOR mothers, there is a higher proportion of [*Prevotella*] at 4 weeks and 32 weeks when compared to mice nursed by NOD mothers. However, NOD-fostered mice have a higher proportion of canonical *Prevotella* at 4 weeks. Differences in *Prevotella* proportions in microbial communities are seen in children, with healthy children having a higher ration of *Prevotella* compared to children with T1D [25]. Interestingly, *Prevotella* is found at a higher level in colorectal cancer patients and patients with Crohn’s disease (CD) compared to healthy controls, indicating that the role of *Prevotella* varies between diseases [26,27]. Through cross-fostering, we have removed some diabetogenic bacteria, while adding bacteria that are associated with diabetic resistance into NOD mice by having them nursed by NOR mothers. We also observed that in mice nursed by NOD or NOR mothers, there was a decrease in *C. Arthromitus* between 4 and 32 weeks. *C. Arthromitus* was identified as a SFB in the gut of arthropods and has recently been shown to play an important role in the maturation of the immune system in the mouse gut [28,29]. This warrants additional investigation because Kriegel *et al.* showed the presence of SFB in female NOD mice correlated with a decrease in the incidence of T1D [30]. It may be possible in the future to use cross-fostering to manipulate levels of *C. Arthromitus* to alter disease incidence. Further research will be required to determine if the higher frequencies of *Firmicutes*, TM7, *Tenericutes*, and *Verrucomicrobia*, as seen in mice nursed by a NOR mother, were critical in the reduction of the incidence of diabetes seen in the male NOD mice nursed by NOR mothers. This data indicates that cross-fostering appears to be a viable method to switch microbiota between strains and potentially protect mice from specific diseases; however, it is also clear that bacteria that are protective from one disease may be promoting a different disease. So, simply cross-fostering to shift microbiota to what is thought to be a healthy state most likely will not protect from all disease and could possibly increase susceptibility to other diseases.

For future microbiota studies, cross-fostering starting at birth appears to be a viable method to induce a shift in microbiota that remains for the entire lifespan of the mice. We are confident that this method could be used for other strains of mice and is not exclusive to the NOD and NOR strains of mice.

## Methods

### Animals

NOD/ShiLtJ mice and NOR/LtJ mice originally obtained from Jackson Laboratory (Bar Harbor, ME, USA) were bred and maintained under specific pathogen-free (SPF) conditions. NOD (*n* = 8) and NOR (*n* = 8) mice were used for experiment 1, and the experiment was repeated with NOD (*n* = 12) and NOR (*n* = 16) mice (experiment 2). All animals were housed in Thoren Isolator ventilated racks (Hazelton, PA, USA). All caging, bedding, and food were sterilized prior to use. Both NOD and NOR mice were put on acidified water within a pH range of 3 to 3.5. Water was acidified using 1 N HCl. Mice were fed autoclaved NIH-31 rodent diet (Harlan Teklan, Madison, WI, USA) *ad libitum*. The Institutional Care and Use Committee of the University of Alabama at Birmingham approved all experiments. A detailed list of our facility’s SPF conditions can be accessed at http://www.uab.edu/research/administration/offices/ARP/ComparativePathology/SupportServices/Pages/HealthSurveillance.aspx.

### Cross-fostering

Breeding pairs of NOD and NOR mice were simultaneously set up when individual mice reached approximately 6 weeks of age. Only pups born to NOD and NOR breeding pairs within 48 h of each other were used for cross-fostering. After the birth of both NOD and NOR litters, half of each litter was removed and put with the mother of the opposite strain. The other half of each litter remained with the birth mother. Litters then contained pups born both to that nursing mother and pups from the opposite NOD or NOR strain (Figure 1). Fostered pups were marked daily with a sharpie on the back of the neck until their ears were able to be clipped (approximately 7 days) for identification purposes. The pups were nursed by their respective mothers until weaning. At weaning, pups were separated based on sex, but not strain, and feces was collected from pups and mothers and stored at −20°C until analysis. When the study ended at 32 weeks, feces was again collected from the previously cross-fostered and control mice, and microbial DNA was isolated from mouse fecal material.

### Sample preparation, sequencing, and analysis

Fecal DNA was isolated using a ZR Fecal DNA MiniPrep™ kit (Zymo Research Corporation, Irvine, CA, USA) as previously described [31]. The oligonucleotide primers used for the PCR amplification of the V4 region of the 16S rRNA gene were as follows (Eurofind Genomics, Inc., Huntsville, AL, USA):

Forward V4:

5′AATGATACGGCGACCACCGAGATCTACACTATGGTAATTGTGTGCCAGCMGCCGCGGTAA 3′; and

Reverse V4:

5′CAAGAGAAGACGGCATACGAGATNNNNNNAGTCAGTCAGCCGGACTACHVGGGTWTCTAAT3′.

For PCR reactions, the conditions were as follows: 10 μL of 5× Reaction Buffer; 1.5 μL (200 μM) of each of the dNTPs; 2 μL (1.5 μM) of each of the primers; 1.5 μL (5 U) of the ‘LongAmp’ enzyme kit (cat # E5200S; New England Biolabs, Ipswich, MA, USA); 30 μL 2 to 5 ng/μL of the Template DNA prepared using the Fecal DNA Isolation kit with the concentration of DNA; 3 μL of H_2_O to a total reaction volume of 50 μL. The PCR cycling parameters were initial denaturation 94°C 1 min; 32 cycles of amplification in which each cycle consisted of 94°C 30 s, 50°C 1 min, 65°C 1 min; followed by an extension step at 65°C for 3 min and a hold at 4°C. Following PCR, the entire PCR reaction was electrophoresed on a 1.0% (*w*/*v*) agarose/Tris-borate-EDTA agarose gel. The PCR product (approximately 380 bp predicted product size) was visualized by UV illumination. The DNA band was excised with a sterile scalpel and purified from the agarose using QIAquick Gel Extraction Kit according to manufacturer’s instructions (#28704; Qiagen, Valencia, CA, USA). The samples were quantitated using Pico Green and adjusted to a concentration of 4 nM [31].

The 16S rDNA V4 region analysis of fecal microbiota was performed as described by Kumar *et al* [31]. Two hundred fifty-one base paired-end sequencing of the amplicons was done using an Illumina MiSeq instrument. FASTQ conversion of the raw data files was performed following de-multiplexing using MiSeq Reporter. Quality assessment of the FASTQ files was performed using FASTQC (http://www.bioinformatics.babraham.ac.uk/projects/fastqc/), and then quality filtering was done using the FASTX toolkit (http://hannonlab.cshl.edu/fastx_toolkit/). Due to low quality of single base at 3′ ends of the read, the last base was trimmed from the 3′ end of all reads before merging using ‘fastq_mergepairs’ command of USEARCH [32]. Any merged read with an average base quality Q score of <20 was discarded.

Sequencing was performed at the UAB Heflin Center for Genomic Sciences, with an average of 83,354 reads per sample. Microbiome amplicon libraries were analyzed using the Quantitative Insight into Microbial Ecology (QIIME) suite version 1.7 [33,34]. For analysis, we used a wrapper for QIIME called QWRAP. Analysis with QWRAP was performed as previously described [31]. Prior to analysis with QWRAP, we merged the overlapping forward and reverse reads using the fastq_mergepairs tool from the USEARCH package [32]. Read pairs with more than 5% mismatches were discarded. This QC method handled the issue of low-quality read tails and replaced the QC metrics found in [21]. Version 13.8 of the Greengenes 16S rRNA database was used for taxanomical assignment of OTUs at an 80% confidence threshold. Proportion levels of the top 50 OTUs at the genus levels can be found in Additional file 4. Output for QWRAP includes taxa summary tables (frequency of all given taxa by sample, by taxanomic level), alpha diversity measurements (chao1, PD whole tree, Shannon, Simpson), and distance matrices and principle coordinate analysis plots for beta diversity.

### Diabetes incidence

Mice were monitored weekly by measuring urine glucose using Diastix® (Bayer, Leverkusen, Germany) starting at 8 weeks of age. Following a positive urine test, a blood glucose test was performed the next day using the OneTouch® Blood Glucose Meter (OneTouch, Greenwood Village, CO, USA). Diabetes was defined as a positive urine test followed by blood glucose test of 200 mg/dL or greater.

### Statistical analysis

Statistical analyses of differences between groups were performed using QIIME’s built-in stats packages. To determine overall differences between the microbiomes of different groups, we tested for significant differences (*P* < 0.05) in beta diversity using compare_categories.py using the PERMANOVA test. The distance matrices used for this test were the same used earlier in the pipeline for PCoA plots, generated by beta_diversity.py using both the weighted and unweighted unifrac metrics. To identify differences between groups at the taxanomical level, we tested for significant differences between groups in the average proportion for each taxon and in the presence/absence of each taxon using otu_category_significance.py with ANOVA and g_test, respectively. The paired *T* test was used for comparisons of taxa proportions between two different time points. Due to the large number of tests (one per taxon), a *P* value was considered significant if it was <0.05 after FDR correction. The taxa summary tables used for these analyses, generated by summarize_taxa.py, were filtered for OTUs with a frequency of less than 0.0005%.

## Availability of supporting data

The data sets supporting the results of this article are available in the NCBI Sequence Read Archive, BioProject is PRJNA277975 (accession # SRP056122) (http://www.ncbi.nlm.nih.gov/bioproject/?term=PRJNA277975).

## Additional files

Additional file 1:
**Weighted unifrac distance matrix showing significance during clustering.** Clustering by nursing mother was also significant when using the weighted unifrac distance matrix (*P* = 0.002 for both nursing mother and age). Additional file 2:
**Top 50 OTUs.** Top 50 OTUs at the genus level in mice nursed by a NOD or NOR mothers at 4 weeks and 32 weeks of age. Additional file 3:
**Grouping of fecal bacterial groups from mice nursed by a NOD or NOR mother.** Grouping of fecal bacterial groups from mice nursed by a NOD or NOR mother, based on caging after weaning. PCoA plots were generated from bacterial DNA that was isolated from mouse fecal material, and the V4 segment of the 16S rRNA gene was amplified from fecal pellets from mice nursed by NOD and NOR mothers (as indicated at the top of the PCoA plot). There was no significant difference in β-diversity between cages nursed by NOD mothers or between cages nursed by NOR mothers. Additional file 4:
**Alpha diversity.** Alpha diversity within samples from mice nursed by a NOD or NOR mother at 4 weeks and 32 weeks of age. There was no significance within samples.

## Abbreviations

CD: Crohn’s disease
FDR: false discovery rate
GI: gastrointestinal
IBD: inflammatory bowel disease
NOD: non-obese diabetic
NOR: non-obese diabetic-resistant
OTU: operational taxonomic unit
PCoA: principal component of analysis
SFB: segmented filamentous bacteria
T1D: type 1 diabetes
TLR: toll-like receptor
