# Supplementary figures and images for: Cross-fostering immediately after birth induces a permanent microbiota shift that is shaped by the nursing mother
Source: Microbiome. 2015 Apr 25;3:17. doi: 10.1186/s40168-015-0080-y (PMC4427954; doi:10.1186/s40168-015-0080-y)

## Slide 1
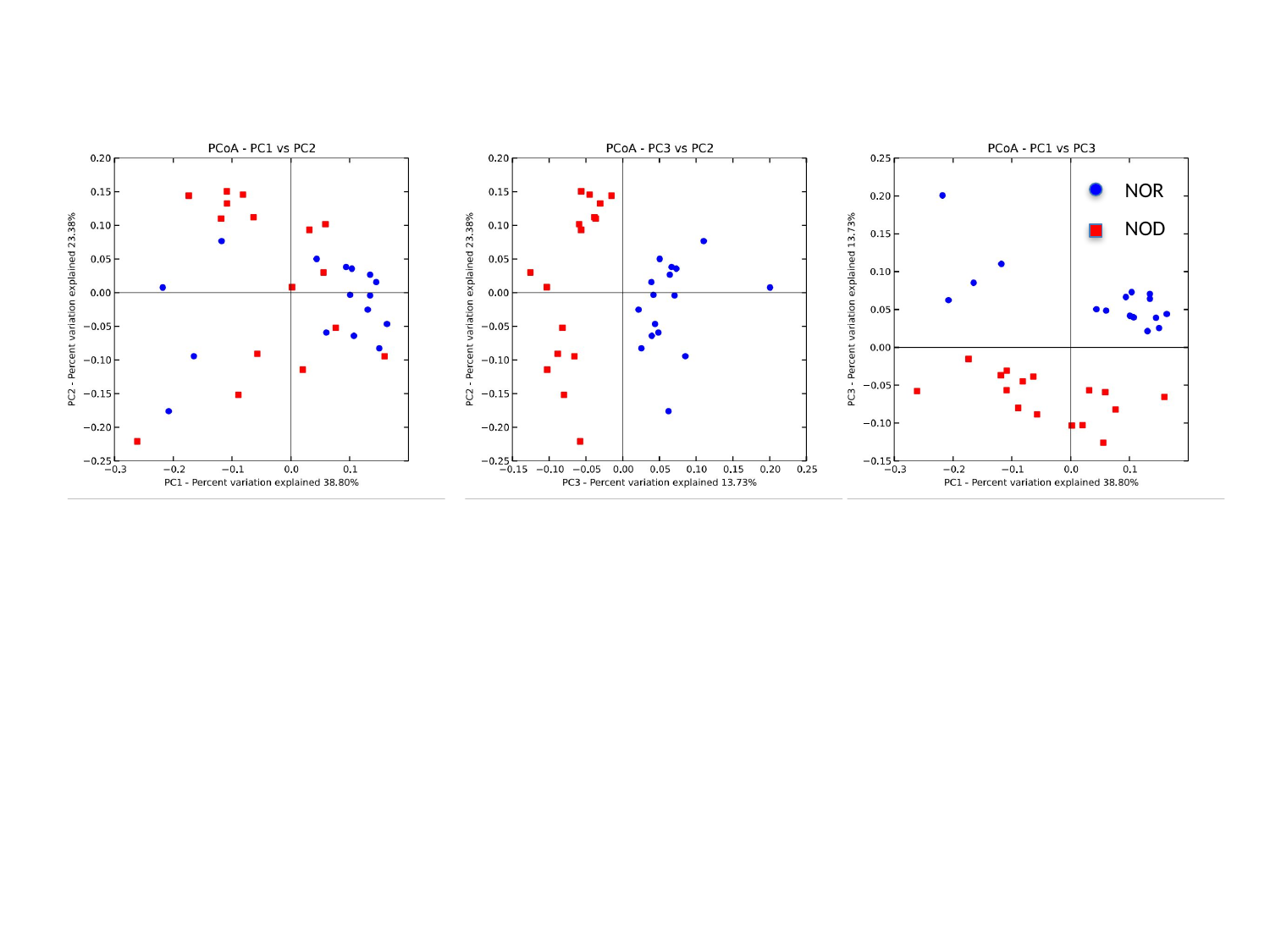

NOR
 NOD

Supplement: Additional file 1: — Weighted unifrac distance matrix showing significance during clustering. Clustering by nursing mother was also significant when using the weighted unifrac distance matrix (P = 0.002 for both nursing mother and age). [file 40168_2015_80_MOESM1_ESM.pptx]

## Slide 1
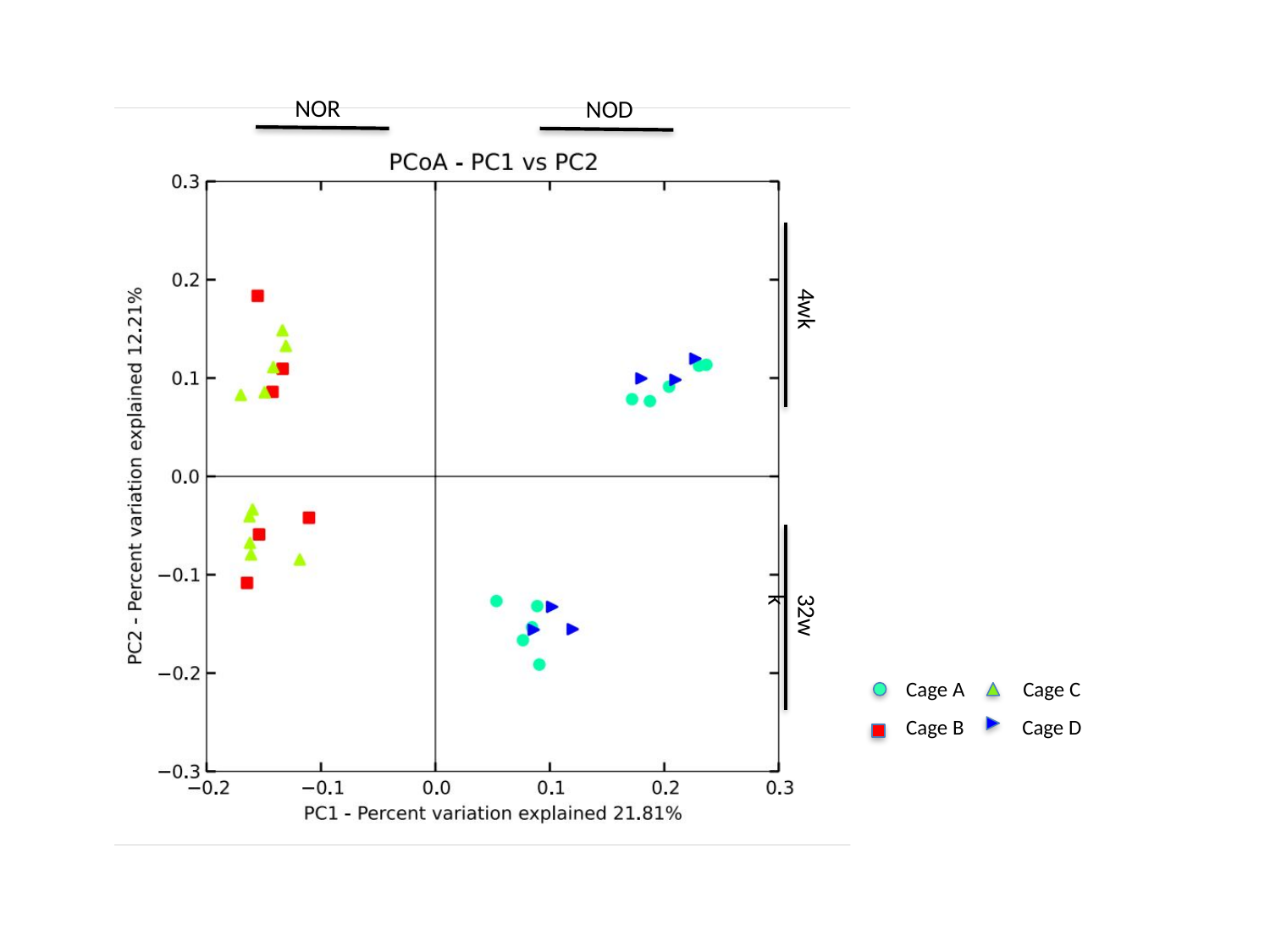

NOR
NOD
4wk
32wk
 Cage A Cage C
 Cage B Cage D

Supplement: Additional file 3: — Grouping of fecal bacterial groups from mice nursed by a NOD or NOR mother. Grouping of fecal bacterial groups from mice nursed by a NOD or NOR mother, based on caging after weaning. PCoA plots were generated from bacterial DNA that was isolated from mouse fecal material, and the V4 segment of the 16S rRNA gene was amplified from fecal pellets from mice nursed by NOD and NOR mothers (as indicated at the top of the PCoA plot). There was no significant difference in β-diversity between cages nursed by NOD mothers or between cages nursed by NOR mothers. [file 40168_2015_80_MOESM3_ESM.pptx]
