# Supplementary material for: Cross-fostering immediately after birth induces a permanent microbiota shift that is shaped by the nursing mother
Source: Microbiome. 2015 Apr 25;3:17. doi: 10.1186/s40168-015-0080-y (PMC4427954; doi:10.1186/s40168-015-0080-y)
Supplement: Additional file 2: — Top 50 OTUs. Top 50 OTUs at the genus level in mice nursed by a NOD or NOR mothers at 4 weeks and 32 weeks of age. [file 40168_2015_80_MOESM2_ESM.docx]

| **Mouse** | **chao1** | **observed_species** | **PD_whole_tree** | **shannon** | **simpson** |
| --- | --- | --- | --- | --- | --- |
| **R-R1 4wk** | 896 | 725 | 34.72 | 6.03 | 0.96 |
| **R-R2 4wk** | 1256 | 890 | 40.60 | 5.60 | 0.95 |
| **R-R3 4wk** | 1155 | 862 | 39.78 | 6.15 | 0.97 |
| **R-R4 4wk** | 1172 | 903 | 41.46 | 5.12 | 0.92 |
| **R-D1 4wk** | 1084 | 813 | 37.46 | 4.83 | 0.90 |
| **R-D2 4wk** | 1113 | 810 | 38.40 | 4.99 | 0.91 |
| **R-D3 4wk** | 1207 | 831 | 38.83 | 5.22 | 0.93 |
| **R-D4 4wk** | 1372 | 972 | 41.39 | 5.58 | 0.94 |
| **D-R1 4wk** | 983 | 718 | 33.02 | 5.36 | 0.95 |
| **D-R2 4wk** | 1288 | 819 | 36.97 | 5.71 | 0.96 |
| **D-R3 4wk** | 884 | 683 | 32.66 | 5.20 | 0.95 |
| **D-R4 4wk** | 1129 | 836 | 36.44 | 5.53 | 0.96 |
| **D-D1 4wk** | 1124 | 793 | 34.74 | 5.40 | 0.95 |
| **D-D2 4wk** | 1279 | 891 | 38.10 | 5.26 | 0.94 |
| **D-D3 4wk** | 1111 | 812 | 35.27 | 4.95 | 0.92 |
| **D-D4 4wk** | 1091 | 714 | 33.34 | 5.63 | 0.96 |
| **R-R1 32wk** | 1223 | 969 | 39.32 | 4.56 | 0.87 |
| **R-R2 32wk** | 1357 | 1053 | 43.63 | 4.96 | 0.90 |
| **R-R3 32wk** | 1389 | 1035 | 42.39 | 4.93 | 0.91 |
| **R-R4 32wk** | 1361 | 991 | 43.24 | 6.47 | 0.97 |
| **R-D1 32wk** | 1255 | 966 | 41.07 | 5.33 | 0.93 |
| **R-D2 32wk** | 1272 | 913 | 37.92 | 4.10 | 0.84 |
| **R-D3 32wk** | 1296 | 981 | 41.54 | 5.59 | 0.90 |
| **R-D4 32wk** | 1554 | 1113 | 45.21 | 5.24 | 0.92 |
| **D-R1 32wk** | 1197 | 792 | 36.59 | 5.34 | 0.93 |
| **D-R2 32wk** | 1402 | 984 | 41.33 | 5.14 | 0.93 |
| **D-R3 32wk** | 1389 | 880 | 37.51 | 4.65 | 0.87 |
| **D-R4 32wk** | 1370 | 967 | 40.75 | 5.18 | 0.94 |
| **D-D1 32wk** | 1094 | 818 | 35.71 | 4.85 | 0.92 |
| **D-D2 32wk** | 917 | 680 | 32.91 | 4.43 | 0.80 |
| **D-D3 32wk** | 1121 | 759 | 35.37 | 5.20 | 0.94 |
| **D-D4 32wk** | 1439 | 1129 | 43.22 | 5.06 | 0.91 |
